# Health and Economic Impacts of Increased Brown Rice Consumption on Type 2 Diabetes in Japan: A Simulation Study, 2019–2029

**DOI:** 10.3390/nu17030532

**Published:** 2025-01-31

**Authors:** Nayu Ikeda, Miwa Yamaguchi, Nobuo Nishi

**Affiliations:** 1 National Institute of Health and Nutrition, National Institutes of Biomedical Innovation, Health and Nutrition, 3-17 Senriokashimmachi, Settsu, Osaka 566-0002, Japan; myamaguchi@nibiohn.go.jp (M.Y.); nishi.nobuo.24@slcn.ac.jp (N.N.); 2 Graduate School of Public Health, St Luke’s International University, 3-6-2 Tsukiji, Chuo-ku, Tokyo 104-0045, Japan

**Keywords:** brown rice, whole grains, type 2 diabetes, Markov model, healthcare costs

## Abstract

Background/Objectives: Whole grain consumption is recognized as a key component of healthy diets, offering protection against non-communicable diseases, including type 2 diabetes (T2D). However, in Japan, whole grain intake remains low, with brown rice—a traditional whole grain—underutilized despite its demonstrated health benefits. This study aimed to explore the health and economic impacts of increasing brown rice consumption among Japanese adults aged 40–79 years. Methods: Using a discrete-time Markov cohort macro-simulation model, we projected the effects of replacing 30% and 80% of mean white rice consumption with brown rice over 10 years. Input parameters were based on published epidemiological data and national healthcare expenditures. Key outcomes included cumulative T2D incidence, all-cause mortality, and associated healthcare costs. Results: Increasing brown rice consumption could prevent 1.3–3.4% of new T2D cases, avert 0.01–0.02% of all-cause deaths, and save from USD 31.3 million to USD 80.5 million in healthcare costs. Sensitivity analyses revealed that the relative risk of T2D associated with brown rice intake had the greatest influence on variabilities in projected cost savings. Conclusions: Although the projected reductions in T2D and healthcare costs were modest, the findings highlight the potential of increased brown rice consumption to alleviate the economic and public health burden of T2D in Japan. Incorporating brown rice into dietary patterns may support multidisciplinary lifestyle approaches for T2D prevention. Further research is warranted to explore long-term care costs for the management of complications and the benefits of other whole grains in the Japanese diet.

## 1. Introduction

Whole grains, including brown rice, millet, oats, rye, and whole wheat bread, are nutritionally superior to refined grains because they retain their hulls and germ, which are rich in dietary fiber, vitamins, and minerals. This nutritional advantage suggests that whole grains play a crucial role in preventing non-communicable diseases. Consequently, whole grain consumption is widely recommended as a key component of sustainable, healthy [1], and planetary health diets [2].

Many countries, including the United States, promote the inclusion of whole grains in healthy eating patterns. For instance, the dietary guidelines of the United States Department of Agriculture and the United States Department of Health and Human Services recommend that at least half of all grain consumption should consist of whole grains [3]. Health economic evaluations in countries such as Australia and the United States have highlighted the potential benefits of increased whole grain intake in reducing the risk of type 2 diabetes (T2D), cardiovascular disease, and certain cancers [4,5,6]. Furthermore, a Markov model simulation conducted in Finland demonstrated that higher whole grain consumption could decrease T2D incidence, reduce healthcare costs, and enhance quality-adjusted life years [7]. With T2D affecting 537 million adults aged 20–79 globally and projections indicating a 46% increase to 783 million by 2045 [8], addressing modifiable risk factors such as inadequate whole grain intake is essential for the growing burden on healthcare systems.

Despite the well-established benefits of whole grains, their consumption remains insufficient in Japan. In 2010, the global average daily intake of whole grains was 38 g, while Japan’s intake was markedly lower, at only 8 g [9]. Among Japanese adults, inadequate whole grain consumption is the third leading dietary factor contributing to the national burden of disease, following excessive salt intake and insufficient fruit consumption [10]. Given Japan’s aging population and declining birth rate, promoting whole grain consumption could be an effective strategy to improve healthy life expectancy and control rising healthcare costs. However, no national guidelines currently promote whole grain intake [11] and no health economic evaluations have been conducted to assess the potential benefits of such an initiative in the country.

Rice is the primary staple food in Japan, accounting for approximately 40% of total consumption (39% in summer and 40% in winter), followed by bread (21% in summer and 16% in winter) and noodles (15% in summer and 12% in winter) [12]. Brown rice, widely recognized as a whole grain in Japan, has been associated with a reduced risk of T2D [13,14,15]. However, only 2% of Japanese adults regularly consume brown rice, with the national median daily intake remaining at zero since 2012 [16]. Given the widespread recognition of brown rice as a whole grain and the relative ease of incorporating brown rice into traditional Japanese diets compared to other whole grain products, this study aimed to develop a simulation model to evaluate the health and economic benefits of increasing brown rice consumption in Japan. Specifically, the model will estimate the impact of replacing white rice with brown rice in the adult population’s diet on T2D incidence and the associated savings in national healthcare expenditures.

## 2. Materials and Methods

### 2.1. Modeling Framework

We modeled the effects of increased brown rice consumption on T2D incidence and national healthcare expenditures in the Japanese adult population. T2D was defined using codes from the International Classification of Diseases, 10th Revision. Notably, we developed a discrete-time Markov cohort macro-simulation model using TreeAge Pro Healthcare 2024 (TreeAge Software, Williamstown, MA, USA) [17]. The Strengthening the Reporting of Observational studies in Epidemiology (STROBE) checklist was used to ensure the transparency and completeness of reporting (Table S1) [18].

We simulated a closed cohort of the total population aged 40 to 79 years in Japan in 2019 over 10 years. Adults under 40 were excluded due to the low incidence of T2D in this age group in Japan [10]. We selected 2019 as the base year for the simulation because it was the most recent year with complete data for all input parameters described below. The time horizon was limited to 10 years to ensure robust results while minimizing the impact of potential long-term social changes. The simulation period was divided into annual cycles and conducted from the perspective of the health sector. We stratified the Markov model by sex and 10-year age groups and applied a half-cycle correction.

**Figure 1:**
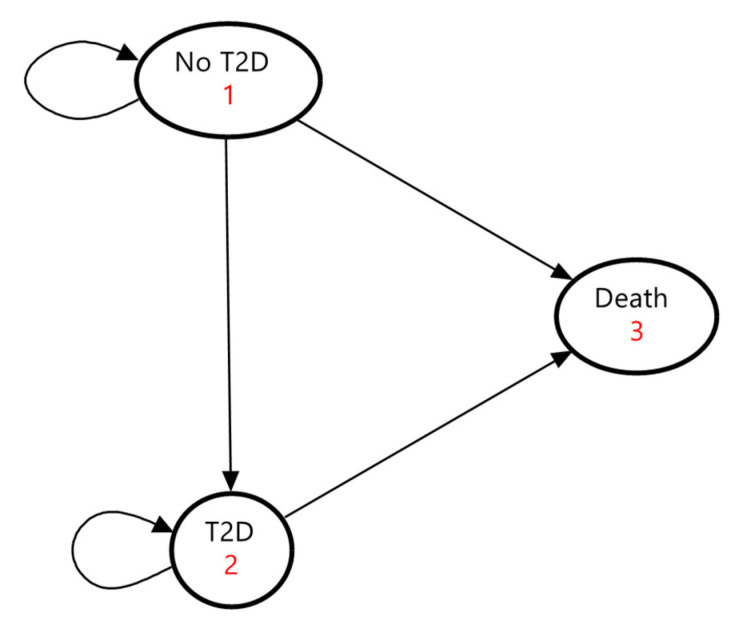
State transition diagram of the Markov model. Ovals represent the three health states. Direct arrows represent directions of transitions of the cohort between health states. Circular arrows indicate the cohort remaining in each health state. Each arrow has a transition probability. T2D, type 2 diabetes.

The model incorporated three mutually exclusive health states as follows: no T2D, T2D, and death (Figure 1). The “no T2D” state represented individuals who had never been diagnosed with T2D, while the “T2D” state represented individuals diagnosed with T2D. The “death” state was an absorbing terminal state. T2D-related complications were not included in the model due to insufficient data on their epidemiology and associated national healthcare costs in Japan.

At the start of the Markov process, the cohort was divided into two states, “no T2D” and “T2D”, based on the prevalence of T2D. Each year, individuals in the cohort transitioned among the three health states based on annual transition probabilities. These probabilities were determined using T2D incidence, all-cause mortality rates, the relative risk of T2D associated with brown rice consumption, and hazard ratios for all-cause mortality among individuals with T2D. Individuals without T2D could either develop T2D, die from all causes, or remain in the “no T2D” state. Once they transitioned to the “T2D” state, they could not return to the “no T2D” state. Individuals diagnosed with T2D could either remain in the same condition or die from all causes.

### 2.2. Scenarios

We examined the following two scenarios for increasing brown rice consumption at the population level: replacing 30% (Scenario 1) and 80% (Scenario 2) of the average white rice consumption with brown rice over 10 years. To assess the potential health gains and savings in national healthcare expenditures, we defined the base case scenario as the reference, where mean brown rice consumption remained at the 2019 level (0 g per day). This status quo scenario was selected because both the mean and median brown rice intake in Japan have remained virtually unchanged at 0 g across all sex and age groups since 2012 [16]. To model changes in mean brown rice intake for each scenario, the baseline values for all sex and age groups were deliberately set to a sufficiently small level of 1 g. Figure S1 illustrates the mean brown rice intake to be attained over time under each scenario. The health and economic effects of increased brown rice consumption were evaluated by comparing the projected incidence, mortality, and national health expenditures between the base case scenario and each of the two scenarios with increased brown rice intake.

### 2.3. Input Parameters

We obtained data for the input parameters from national databases and the published scientific literature (Table 1). The baseline average intake of white rice (Table S2) was estimated using individual-level dietary intake records based on food items from the Japan National Health and Nutrition Survey conducted in 2019 [19]. We obtained official approval to access individual-level data in accordance with the Statistics Act [20]. Ethical review was not required for this study, as the use of the survey data is exempt under the Ethical Guidelines for Medical and Biological Research Involving Human Subjects [21]. The survey employed a stratified two-stage cluster sampling design to ensure a nationally representative sample of the non-institutionalized Japanese population [19]. Household representatives reported food intake for each household member using a 1-day semi-weighted household dietary record. Each food item was coded according to the food codes from the 2015 edition of the Standard Tables of Food Composition in Japan [22].

To estimate the mean white rice intake based on sex and age group at baseline, we analyzed data from a sample of 3605 participants aged 40 to 79 years (1705 men and 1900 women) after excluding 618 participants (14.6%; 302 men and 316 women) with missing food intake data. To classify white rice consumption, we included eight non-glutinous rice items listed in Table S6, excluding glutinous rice because it is primarily used in traditional sweets and special occasions. Weight change factors from Table S6 were applied to convert the weights of cooked rice items to their respective dry equivalents.

The transition probability from the “no T2D” state to the “T2D” state was calculated by multiplying T2D incidence among people without T2D by the relative risk associated with increased brown rice consumption. Similarly, we determined the probability of transitioning from the T2D state to death by multiplying all-cause mortality by the hazard ratios for all-cause mortality in individuals with T2D.

For national healthcare expenditures, we assigned the sum of the costs of inpatient and outpatient care and drug prescriptions to the T2D state. We converted national healthcare expenditures from Japanese yen to United States dollars (USD) according to the 2019 annual average exchange rate of JPY 109.01 per USD, as published by the International Monetary Fund [27]. We discounted these expenditures at 2% annually, according to the guidelines for economic evaluation of healthcare technologies in Japan [28].

### 2.4. Sensitivity Analyses

We conducted multiple deterministic one-way sensitivity analyses to assess the impact of uncertainty in model input parameters on the projected savings in national healthcare expenditures. The parameters tested in these analyses included the discount rate (ranging from 0% to 4%), the mean white rice intake, and the incidence, prevalence, and relative risk of T2D (using the lower and upper bounds of the 95% confidence intervals in Table 1, Tables S2 and S3).

## 3. Results

### 3.1. Projected Base-Case Incidence, Mortality, and National Healthcare Expenditures

Table 2 shows the projected cumulative incidence, mortality rate, and national healthcare expenditures for T2D at the end of the 10-year simulation period under the base case scenario. If the average daily brown rice intake of 0 g in 2019 were sustained over the decade, the cohort would experience a total of 3,785,678 new cases of T2D, ranging from 258,847 among women in their 40s to 695,282 among men in their 60s. The cumulative number of deaths from all causes would reach 5,070,213 (7.6%), with figures ranging from 86,793 (0.9%) among women in their 40s to 1,996,749 (25.7%) among men in their 70s. The cumulative discounted national healthcare expenditures would total USD 15.8 billion, ranging from USD 168.6 million for women in their 40s to USD 4.4 billion for men in their 70s.

### 3.2. Health Gains from Increased Consumption of Brown Rice

Table 3 presents the cumulative incidence and mortality averted by replacing white rice with brown rice compared to the base case scenario. For the entire cohort, the total number of T2D cases averted would be 49,413 (1.3%) in Scenario 1 and 129,367 (3.4%) in Scenario 2. The relative reductions in T2D incident cases were greater in men (1.5% and 4.0%) than in women (1.0% and 2.7%) for both scenarios. The cumulative number of all-cause deaths averted in the cohort was projected to be 506 (0.01%) for Scenario 1 and 1080 (0.02%) for Scenario 2. The relative reductions in deaths were nearly identical between men and women, with 0.01% for Scenario 1 and 0.02% for Scenario 2.

### 3.3. National Healthcare Expenditures Saved by Increased Consumption of Brown Rice

Table 4 summarizes the projected cumulative savings in national healthcare expenditures resulting from the prevention of T2D due to increased brown rice consumption. The total cumulative savings for the entire cohort would amount to USD 31.3 million (0.2%) for Scenario 1 and USD 80.5 million (0.5%) for Scenario 2. Savings varied by sex and age group, ranging from USD 0.3 million for women in their 40s in Scenario 1 to USD 28.7 million for men in their 60s in Scenario 2. The relative reductions in national healthcare expenditures were slightly greater for men (0.2% and 0.6%) than for women (0.1% and 0.3%) under both scenarios.

### 3.4. Sensitivity Analyses

**Figure 2:**
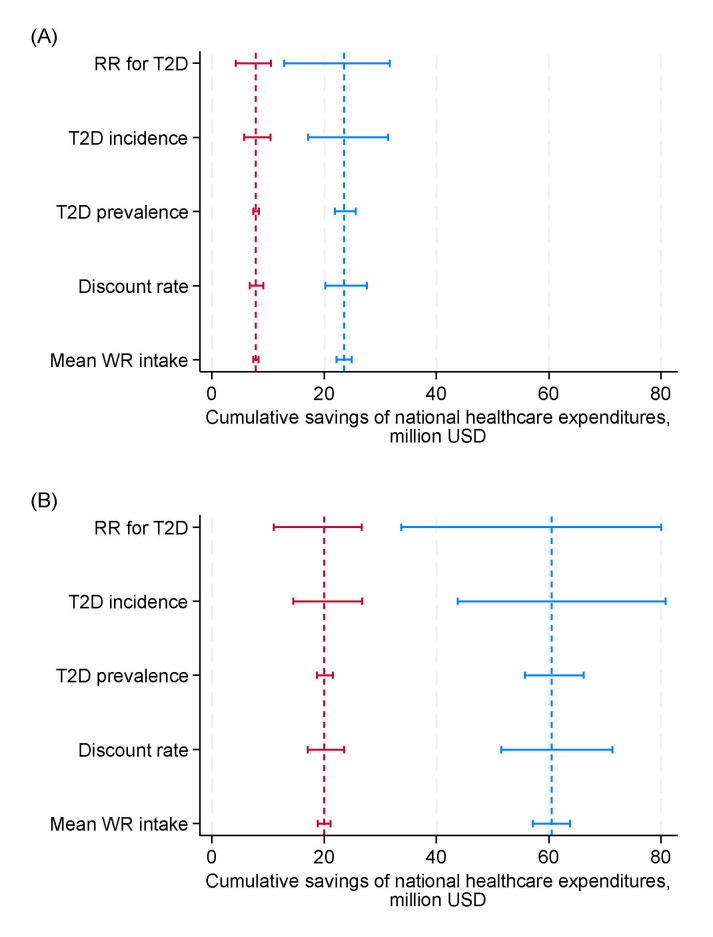
Results of one-way sensitivity analyses on projected cumulative savings in national healthcare expenditures for type 2 diabetes by replacing (**A**) 30% and (**B**) 80% of mean daily white rice intake with brown rice intake over 10 years from 2019 in a closed cohort of population aged 40–79 years in 2019. Blue, men; red, women. Range plots with spikes indicate uncertainty ranges, and dashed lines represent expected values. RR, relative risk; T2D, type 2 diabetes; WR, white rice.

Figure 2 presents the results of the one-way sensitivity analyses for the entire cohort. The greatest uncertainty in the projected cumulative savings in national healthcare expenditures under Scenarios 1 and 2 was associated with the relative risk of T2D for increased brown rice consumption, followed by uncertainty in T2D incidence.

## 4. Discussion

This study is the first to assess the health and economic impacts of promoting brown rice consumption as a public health strategy to prevent T2D in Japan. Our simulation results suggest that replacing 30% to 80% of average white rice intake with brown rice over 10 years could reduce T2D incidence by 1–3%. This finding aligns with existing evidence that brown rice consumption improves glycemic control and lowers T2D risk due to its higher nutrient content [13,14,15]. The benefits were more pronounced in men, likely due to their higher baseline T2D rates. Although the reduction in all-cause mortality was small, preventing 0.01–0.02% of deaths over a decade suggests that increased brown rice consumption could make a modest contribution to improving population health. Moreover, incorporating brown rice into dietary patterns supports contemporary lifestyle approaches that focus on addressing modifiable risk factors, such as poor diets, through multidisciplinary strategies for preventing noncommunicable diseases [29].

From an economic perspective, the modeled scenarios could reduce national healthcare costs by 0.2–0.5%. Although these savings may appear modest, they indicate the potential of brown rice consumption to alleviate the economic burden of non-communicable diseases such as T2D. The savings were more pronounced among men, reflecting their higher T2D incidence and healthcare expenditures. These findings underscore the cost-effectiveness of promoting brown rice as a preventive measure against T2D in Japan. Our results align with previous research from Finland, which demonstrated both the health and economic benefits of increasing whole grain consumption for T2D prevention in adults [7].

Incorporating brown rice into dietary patterns could encourage the consumption of plant-based foods, which are essential for a healthy and environmentally sustainable diet [16]. Brown rice consumers typically also eat more vegetables and legumes, reflecting a preference for plant-based nutrition. Additionally, brown rice consumption is associated with higher intakes of essential nutrients that may support balanced nutrition by encouraging the consumption of other plant-based foods.

However, increasing brown rice consumption faces challenges in Japan, where only 2% of adults incorporate brown rice into their diet [16]. This mirrors the low consumption rates observed in the United States in the 2000s [30,31], highlighting a broader issue of low whole grain intake in Japan. Our simulation highlights the need for targeted nutrition policies to promote whole grain consumption, as Japan currently lacks specific policies advocating for whole grains [11], despite their inclusion in dietary guidelines in the United States and other countries [32,33].

The adoption of brown rice is limited by its longer cooking time, distinct texture, and flavor, which may not appeal to everyone, especially in larger households. Barriers to acceptance include the limited awareness of its nutritional benefits and sensory attributes [34,35,36,37], as well as practical challenges like market availability and cost. Addressing these barriers through health education, school lunch programs, and efforts to reduce costs could improve acceptance. Technological advancements, such as improved rice varieties and specialized rice cookers, could also make brown rice more appealing.

A 2018 survey in the Greater Tokyo Area showed that 7% of respondents consumed brown rice three or more times weekly [38]. While health benefits were widely recognized, negative perceptions about taste, cooking difficulty, and cost persisted. Moreover, many respondents were unaware of the technological advancements such as improved digestibility and texture. Overcoming these obstacles will require a collaborative effort across sectors, including the rice industry and the promotion of processed brown rice and pre-germinated rice [39].

Demographic groups such as women, older adults, urban residents, and those with higher education levels are more likely to consume brown rice [16]. Targeting these groups, particularly through public education and increased availability, could help spread awareness and increase the acceptance of brown rice across broader populations. Special consideration is needed for parents of young children who may be concerned about slower digestion and potential stomach discomfort from brown rice.

Our study has several strengths, including the use of national-level statistics on white rice intake and the integration of epidemiological and healthcare cost data to assess the impact of increased brown rice consumption. Moreover, we conducted deterministic one-way sensitivity analyses to account for uncertainties in our projections. However, the findings were subject to considerable uncertainty, primarily regarding the relative risk of T2D associated with brown rice consumption. Given that current evidence is based on cohort studies in the United States [13,14], further research is needed to quantify the preventive impact of brown rice on T2D more accurately.

Several limitations should be considered when interpreting our results. First, the model assumed immediate effects from increased brown rice consumption without accounting for a gradual phase-in period. Future research should investigate whether the benefits of brown rice consumption on T2D outcomes take longer to materialize. Second, our analysis focused solely on medical expenditures related to T2D and did not include the long-term costs of managing complications like macrovascular and microvascular diseases due to insufficient input data. Since these complications may increase disability and mortality burdens [40], addressing them in future studies is essential. Third, the study was limited to a cohort aged 40–79 years in 2019 and did not account for population changes over the 10-year period, making these results specific to this cohort. Fourth, the simulation’s time horizon was restricted to 10 years to minimize the influence of long-term social changes. Extending the simulation to 20 or 30 years in future research would provide additional insights, provided that adequate input data are available. Fifth, the analysis modeled simple scenarios of replacing a portion of average white rice intake with brown rice, without considering the effects of educational campaigns and their associated costs. Thus, the findings cannot address the potential influence of such campaigns on the acceptance of brown rice in Japan. Finally, while this study focused on brown rice as a widely recognized whole grain in Japan, future research should investigate the health and economic effects of increasing the consumption of other whole grain products, such as oats and rye.

## 5. Conclusions

Increasing brown rice consumption offers a promising strategy for preventing T2D in Japan, potentially reducing both T2D incidence and healthcare costs. Despite challenges related to cultural preferences and practical barriers, promoting brown rice as part of a broader shift toward whole grains could improve public health outcomes. Innovations in rice varieties, increased awareness, and the targeted engagement of key demographic groups could enhance acceptance. However, further research is needed to quantify the long-term effects of brown rice intake on T2D prevention. Policymakers should consider integrating brown rice into national dietary guidelines, with a focus on improving access, affordability, and public education in order to reduce barriers and enhance population health.

## Figures and Tables

**Table 1:** Input parameters and data sources used in the simulation model.

Input Parameters	Data Sources	Values
Total population	Population Estimates, 2019 [23]	[Table S2]
Mean white rice intake	National Health and Nutrition Survey in Japan, 2019 [19]	[Table S2]
Prevalence and incidence rates of T2D	Global Burden of Disease Study 2021 [10]	[Table S3]
Mortality rates of all causes	Global Burden of Disease Study 2021 [10]	[Table S3]
Relative risk for T2D associated with replacing white rice intake with brown rice	Meta-analysis of 3 prospective cohorts of health professionals in the United States [14]	0.84 (0.79, 0.91)
Relative risks for all-cause mortality associated with T2D	Meta-analysis of 22 prospective cohorts of the Asia Cohort Consortium [24]	[Table S4]
National healthcare expenditures	Survey on Medical Care Benefit, 2019 [25], Survey on Prescription Drug Expenditure, 2019 [26]	[Table S5]

T2D, type 2 diabetes. Values in parentheses indicate lower and upper bounds of the 95% confidence interval.

**Table 2:** Projected cumulative incidence and national healthcare expenditures for type 2 diabetes and all-cause mortality over 10 years from 2019 in a closed cohort of the population aged 40–79 years in 2019 under the base case scenario of mean brown rice intake remaining at 2019 levels.

Sex, Age (Years)	Population	T2D Incidence		All-Cause Death		National Healthcare Expenditures for T2D, USD
	No.	No.	(%)	No.	(%)	
Men						
40–79	32,797,470	2,064,200	(6.3)	3,354,874	(10.2)	10,061,421,208
40–49	9,373,569	408,411	(4.4)	150,248	(1.6)	495,187,931
50–59	8,160,860	668,028	(8.2)	358,224	(4.4)	1,489,398,961
60–69	7,929,684	695,282	(8.8)	959,652	(12.1)	3,645,371,892
70–79	7,333,357	292,479	(4.0)	1,886,749	(25.7)	4,431,462,425
Women						
40–79	34,158,646	1,721,478	(5.0)	1,715,339	(5.0)	5,709,993,001
40–49	9,146,186	258,847	(2.8)	86,793	(0.9)	168,554,681
50–59	8,116,993	512,116	(6.3)	179,677	(2.2)	568,550,514
60–69	8,301,898	623,188	(7.5)	421,289	(5.1)	1,862,203,904
70–79	8,593,569	327,327	(3.8)	1,027,580	(12.0)	3,110,683,903

T2D, type 2 diabetes.

**Table 3:** Projected cumulative incidence of type 2 diabetes and all-cause deaths prevented by replacing mean white rice intake with brown rice over 10 years from 2019 in a closed cohort of the population aged 40–79 years in 2019.

Sex, Age (Years)	T2D Incidence				All-Cause Death			
	Scenario 1		Scenario 2		Scenario 1		Scenario 2	
	No.	(%)	No.	(%)	No.	(%)	No.	(%)
Men								
40–79	31,742	(1.5)	82,445	(4.0)	384	(0.01)	820	(0.02)
40–49	7117	(1.7)	18,440	(4.5)	27	(0.02)	58	(0.04)
50–59	10,838	(1.6)	28,136	(4.2)	84	(0.02)	179	(0.05)
60–69	9916	(1.4)	25,822	(3.7)	177	(0.02)	377	(0.04)
70–79	3871	(1.3)	10,047	(3.4)	96	(0.01)	206	(0.01)
Women								
40–79	17,671	(1.0)	46,922	(2.7)	121	(0.01)	261	(0.02)
40–49	3088	(1.2)	8164	(3.2)	8	(0.01)	17	(0.02)
50–59	5347	(1.0)	14,208	(2.8)	24	(0.01)	51	(0.03)
60–69	5977	(1.0)	15,916	(2.6)	50	(0.01)	107	(0.03)
70–79	3258	(1.0)	8635	(2.6)	39	(0.00)	85	(0.01)

T2D, type 2 diabetes. Scenario 1: replacement of 30% of mean daily white rice intake with brown rice in 2019 over 10 years. Scenario 2: replacement of 80% of mean daily white rice intake with brown rice in 2019 over 10 years.

**Table 4:** Projected cumulative discounted savings of national healthcare expenditures for type 2 diabetes by replacing mean white rice intake with brown rice over 10 years from 2019 in a closed cohort aged 40–79 years in 2019, compared with the base case scenario.

Sex, Age (Years)	Scenario 1		Scenario 2	
	USD	(%)	USD	(%)
Men				
40–79	23,535,925	(0.2)	60,507,861	(0.6)
40–49	1,328,875	(0.3)	3,200,548	(0.6)
50–59	5,302,819	(0.4)	13,291,622	(0.9)
60–69	11,077,131	(0.3)	28,737,698	(0.8)
70–79	5,827,099	(0.1)	15,277,992	(0.3)
Women				
40–79	7,786,800	(0.1)	19,972,435	(0.3)
40–49	280,164	(0.2)	665,984	(0.4)
50–59	1,222,818	(0.2)	3,037,052	(0.5)
60–69	3,435,131	(0.2)	8,860,798	(0.5)
70–79	2,848,687	(0.1)	7,408,601	(0.2)

Scenario 1: replacement of 30% of mean daily white rice intake with brown rice in 2019 over 10 years. Scenario 2: replacement of 80% of mean daily white rice intake with brown rice in 2019 over 10 years.

## Data Availability

The availability of data analyzed in this study is restricted under the Statistics Act.

## Supplementary Materials

The following supporting information can be downloaded at https://www.mdpi.com/article/10.3390/nu17030532/s1, Figure S1: Mean daily brown rice intake to be achieved between the years 2019 and 2029, by age group and sex; Table S1: STROBE Statement—checklist of items that should be included in reports of observational studies; Table S2: Baseline data on total population and mean white rice intake by age group and sex in Japan, 2019; Table S3: Baseline data on the incidence, prevalence, and mortality of type 2 diabetes mellitus and all-cause mortality, per 100,000, by age group and sex in Japan, 2019, based on estimates from the Global Burden of Disease Study 2021; Table S4: Relative risks for all-cause mortality associated with type 2 diabetes mellitus used in the model, by age group, both sexes; Table S5: Baseline data on national healthcare expenditures for type 2 diabetes mellitus by age group and sex in Japan, 2019, in US dollars; Table S6: Food items and weight change factors.

## Abbreviation

The following abbreviations are used in this manuscript:

T2D	type 2 diabetes
