# Supplementary material for: Health and Economic Impacts of Increased Brown Rice Consumption on Type 2 Diabetes in Japan: A Simulation Study, 2019–2029
Source: Nutrients. 2025 Jan 31;17(3):532. doi: 10.3390/nu17030532 (PMC11821163; doi:10.3390/nu17030532)
Supplement: Supplementary file 1 [file nutrients-17-00532-s001.zip › nutrients-3457239-supplementary.pdf]

Supplementary material

Health and economic impacts of increased brown rice consumption on type 2 diabetes in Japan: a simulation study, 2019–2029

Nayu Ikeda, Miwa Yamaguchi, Nobuo Nishi

Correspondence: Nayu Ikeda, PhD
ikedan@nibiohn.go.jp

Laboratory of Nutrition and Social Science
Center for Nutritional Epidemiology and Policy Research
National Institute of Health and Nutrition
National Institutes of Biomedical Innovation, Health and Nutrition
Settsu, Osaka, Japan

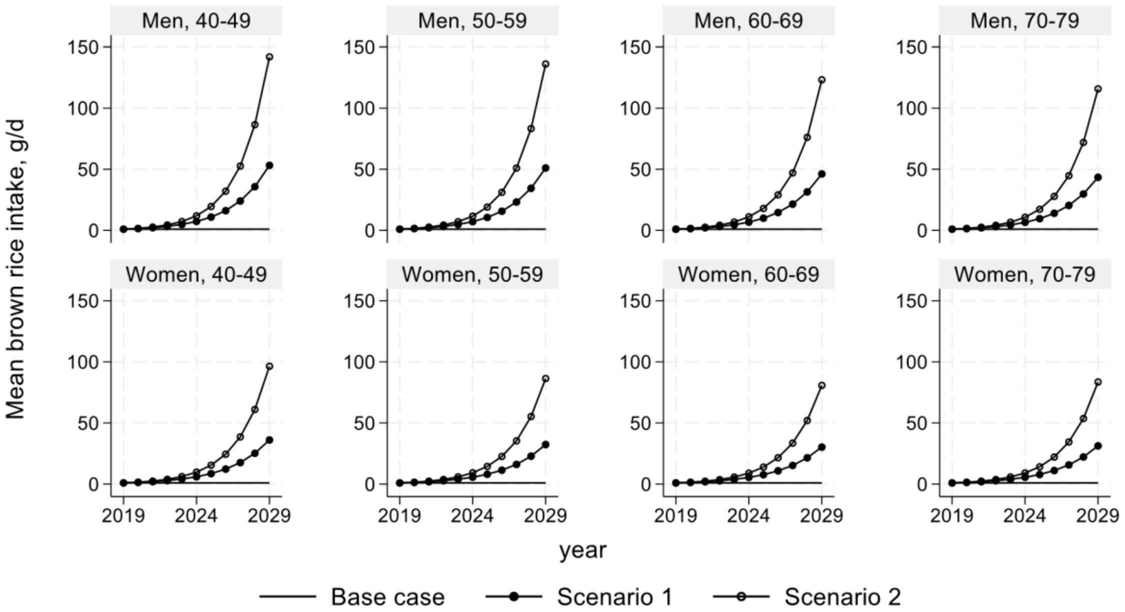

Figure S1. Mean daily brown rice intake to be achieved between the years 2019 and 2029, by age group and sex. Base case refers to sustaining the baseline levels of 2019. Scenario 1: replacement of 30% of mean white rice intake with brown rice in 2019 over 10 years. Scenario 2: replacement of 80% of mean white rice intake with brown rice in 2019 over 10 years.

Table S1. STROBE Statement—checklist of items that should be included in reports of observational studies

	Item No	Recommendation	Page No.
**Title and abstract**	1	(*a*) Indicate the study's design with a commonly used term in the title or the abstract	1
		(*b*) Provide in the abstract an informative and balanced summary of what was done and what was found	1
**Introduction**			
Background/rationale	2	Explain the scientific background and rationale for the investigation being reported	1-2
Objectives	3	State specific objectives, including any prespecified hypotheses	2
**Methods**			
Study design	4	Present key elements of study design early in the paper	2-3
Setting	5	Describe the setting, locations, and relevant dates, including periods of recruitment, exposure, follow-up, and data collection	2-4
Participants	6	(*a*) *Cohort study*—Give the eligibility criteria, and the sources and methods of selection of participants. Describe methods of follow-up	2-3
Variables	7	Clearly define all outcomes, exposures, predictors, potential confounders, and effect modifiers. Give diagnostic criteria, if applicable	3
Data sources/ measurement	8*	For each variable of interest, give sources of data and details of methods of assessment (measurement). Describe comparability of assessment methods if there is more than one group	4
Bias	9	Describe any efforts to address potential sources of bias	2-3
Study size	10	Explain how the study size was arrived at	2, 4
Quantitative variables	11	Explain how quantitative variables were handled in the analyses. If applicable, describe which groupings were chosen and why	4
Statistical methods	12	(*a*) Describe all statistical methods, including those used to control for confounding	4
		(*b*) Describe any methods used to examine subgroups and interactions	3, 4
		(*c*) Explain how missing data were addressed	4
		(*d*) *Cross-sectional study*—If applicable, describe analytical methods taking account of sampling strategy	4
		(*e*) Describe any sensitivity analyses	5
**Results**			
Participants	13*	(a) Report numbers of individuals at each stage of study—eg numbers potentially eligible, examined for eligibility, confirmed eligible, included in the study, completing follow-up, and analysed	4
		(b) Give reasons for non-participation at each stage	NA
		(c) Consider use of a flow diagram	NA
Descriptive data	14*	(a) Give characteristics of study participants (eg demographic, clinical, social) and information on exposures and potential confounders	Tables S1-S4
		(b) Indicate number of participants with missing data for each variable of interest	4
Outcome data	15*	*Cross-sectional study*—Report numbers of outcome events or summary measures	5, 6
Main results	16	(*a*) Give unadjusted estimates and, if applicable, confounder-adjusted estimates and their precision (eg, 95% confidence interval). Make clear which confounders were adjusted for and why they were included	NA
		(*b*) Report category boundaries when continuous variables were categorized	NA
		(*c*) If relevant, consider translating estimates of relative risk into absolute risk for a meaningful time period	NA
Other analyses	17	Report other analyses done—eg analyses of subgroups and interactions, and sensitivity analyses	7
**Discussion**			

Key results	18	Summarise key results with reference to study objectives	7-8
Limitations	19	Discuss limitations of the study, taking into account sources of potential bias or imprecision. Discuss both direction and magnitude of any potential bias	9
Interpretation	20	Give a cautious overall interpretation of results considering objectives, limitations, multiplicity of analyses, results from similar studies, and other relevant evidence	8-9
Generalisability	21	Discuss the generalisability (external validity) of the study results	7-9
**Other information**			
Funding	22	Give the source of funding and the role of the funders for the present study and, if applicable, for the original study on which the present article is based	10

NA, not applicable.

*Give information separately for cases and controls in case-control studies and, if applicable, for exposed and unexposed groups in cohort and cross-sectional studies.

Note: An Explanation and Elaboration article discusses each checklist item and gives methodological background and published examples of transparent reporting. The STROBE checklist is best used in conjunction with this article (freely available on the Web sites of PLoS Medicine at http://www.plosmedicine.org/, Annals of Internal Medicine at http://www.annals.org/, and Epidemiology at http://www.epidem.com/). Information on the STROBE Initiative is available at www.strobe-statement.org.

Table S2. Baseline data on total population and mean white rice intake by age group and sex in Japan, 2019

Sex, age in years	Total population [1]	Mean white rice intake, g/d
Men		
40–49	9,373,569	177.4 (166.4, 188.4)
50–59	8,160,860	170.1 (159.5, 180.7)
60–69	7,929,684	154.0 (144.7, 163.2)
70–79	7,333,357	144.7 (137.1, 152.3)
Women		
40–49	9,146,186	120.5 (112.6, 128.4)
50–59	8,116,993	108.0 (101.5, 114.5)
60–69	8,301,898	101.0 (95.0, 107.0)
70–79	8,593,569	104.4 (98.2, 110.7)

Values in parentheses indicate lower and upper bounds of 95% confidence intervals.

Table S3. Baseline data on the incidence, prevalence, and mortality of type 2 diabetes mellitus and all-cause mortality, per 100,000, by age group and sex in Japan, 2019, based on estimates from the Global Burden of Disease Study 2021 [2]

Sex, age in years	T2D incidence	T2D prevalence	T2D mortality	All-cause mortality
Men				
40–49	448.1 (317.8, 621.6)	7,521.8 (6,361.2, 8,687.1)	1.2 (1.1, 1.2)	142.2 (141.3, 143.2)
50–59	870.8 (662.8, 1,106.2)	13,102.3 (11,262.4, 15,343.9)	3.9 (3.8, 4.1)	380.5 (377.7, 383.4)
60–69	971.1 (750.2, 1,216.8)	21,758.7 (19,027.1, 24,739.1)	9.7 (9.3, 10.1)	1,050.4 (1,043.3, 1,057.9)
70–79	459.6 (329.0, 609.0)	27,430.1 (24,340.9, 30,834.7)	19.4 (18.0, 20.4)	2,560.7 (2,544.7, 2,577.3)
Women				
40–49	288.0 (199.8, 407.7)	4,942.8 (4,159.2, 5,782.0)	0.4 (0.4, 0.4)	87.5 (86.7, 88.5)
50–59	657.5 (494.8, 838.5)	8,927.6 (7,692.6, 10,573.2)	1.0 (1.0, 1.1)	198.8 (196.9, 200.9)
60–69	799.0 (618.5, 1,007.5)	15,937.4 (13,793.4, 18,226.2)	2.7 (2.4, 2.8)	445.1 (441.3, 449.0)
70–79	410.1 (296.9, 542.8)	20,994.9 (18,621.1, 23,728.4)	8.0 (6.6, 8.7)	1,135.7 (1,126.8, 1,145.1)

T2D, type 2 diabetes mellitus. Original estimates by 5-year age groups and sex in the Global Burden of Disease Study were averaged between two adjacent age groups to obtain estimates by 10-year age group and sex. Values in parentheses indicate lower and upper bounds of 95% confidence intervals.

Table S4. Relative risks for all-cause mortality associated with type 2 diabetes mellitus used in the model, by age group, both sexes [3]

Age in years	Hazard ratio (95% confidence interval)
40–49	2.43 (2.08, 2.84)
50–59	2.06 (1.83, 2.31)
60–69	1.87 (1.71, 2.05)
70–79	1.51 (1.40, 1.62)

Table S5. Baseline data on national healthcare expenditures for type 2 diabetes mellitus by age group and sex in Japan, 2019, in US dollars

Sex, age in years	Inpatient [4]	Outpatient [4]	Prescription drug [4]*
Men			
40–49	65,104,817	320,075,275	184,500,399
50–59	107,029,405	599,298,804	272,090,880
60–69	208,951,045	1,084,370,952	444,171,579
70–79	366,115,328	1,450,931,335	512,741,067
Women			
40–49	27,656,604	149,289,472	118,176,373
50–59	52,349,246	284,378,210	184,737,605
60–69	115,853,557	620,072,019	340,480,194
70–79	278,606,768	984,325,456	457,219,820

The expenditures were converted from Japanese yen to US dollar according to the annual average exchange rate in 2019 (109.01 JPY per USD) [5].
* Data on prescription drug for type 2 diabetes mellitus were only available of the total population (approximately 3.4 billion US dollars) in the original data source. To obtain data by age group and sex, the total drug cost was multiplied by proportions of people having type 2 diabetes in each age group and sex out of the total number of people with the disease obtained from the Global Burden of Disease Study [2].

Table S6. Food items and weight change factors for estimating white rice intake. This table presents the food items included in the study with their corresponding weight change factors, obtained from the Standard Tables of Food Composition in Japan, 2015 edition [6]

Food item	Weight change factor
Rice, paddy rice, non-glutinous, well-milled, raw	Not applicable
Rice, short grain, paddy rice, non-glutinous rice, well-milled, “meshi” (cooked rice)	210
Rice, short grain, paddy rice, well-milled, “zengayu” (gruel)	500
Rice, short grain, paddy rice, well-milled, “gobugayu” (diluted gruel)	1,000
Rice, short grain, paddy rice, well-milled, “omoyu” (thin gruel)	1,700
Rice, non-glutinous rice products, quick-cooking rice, regular, raw	210
Rice, non-glutinous rice products, “Onigiri” (rice ball)*	210
Rice, non-glutinous rice products, “Yaki-onigiri” (baked rice ball)**	210

* Without laver and filling; containing 0.5 g of salt per 100 g.

** Containing 6.5 g of common soy sauce per 100 g.

References

1. Statistics Bureau of Japan, Ministry of Internal Affairs and Communications. Current Population Estimates as of October 1, 2019. Availabe online: https://www.stat.go.jp/english/data/jinsui/2019np/index.html (accessed on 9 January 2025).
2. GBD 2021 Risk Factors Collaborators. Global burden and strength of evidence for 88 risk factors in 204 countries and 811 subnational locations, 1990–2021: a systematic analysis for the Global Burden of Disease Study 2021. *The Lancet* **2024**, *403*, 2162-2203, doi:https://doi.org/10.1016/S0140-6736(24)00933-4.
3. Yang, J.J.; Yu, D.; Wen, W.; Saito, E.; Rahman, S.; Shu, X.-O.; Chen, Y.; Gupta, P.C.; Gu, D.; Tsugane, S., et al. Association of Diabetes With All-Cause and Cause-Specific Mortality in Asia: A Pooled Analysis of More Than 1 Million Participants. *JAMA Network Open* **2019**, *2*, e192696-e192696, doi:10.1001/jamanetworkopen.2019.2696.
4. Ministry of Health, Labour and Welfare. Survey on Prescription Drug Expenditure, 2019. Availabe online: https://www.mhlw.go.jp/bunya/iryouhoken/database/zenpan/cyouzai_doukou.html (accessed on 7 January 2025).
5. International Monetary Fund. IMF data access to macroeconomic & financial data. Availabe online: https://data.imf.org/?sk=388dfa60-1d26-4ade-b505-a05a558d9a42 (accessed on 7 January 2025).
6. Ministry of Education, Culture, Sports, Science, and Technology. Standard tables of food composition in Japan - 2015 - (Seventh revised edition). Availabe online: https://www.mext.go.jp/en/policy/science_technology/policy/title01/detail01/1374030.htm (accessed on 9 January 2025).
